# VX Hydrolysis by Human Serum Paraoxonase 1: A Comparison of Experimental and Computational Results

**DOI:** 10.1371/journal.pone.0020335

**Published:** 2011-05-31

**Authors:** Matthew W. Peterson, Steven Z. Fairchild, Tamara C. Otto, Mojdeh Mohtashemi, Douglas M. Cerasoli, Wenling E. Chang

**Affiliations:** 1 The MITRE Corporation, Bedford, Massachusetts, United States of America; 2 Department of Biomedical Engineering, Boston University, Boston, Massachusetts, United States of America; 3 Physiology and Immunology Branch, Research Division, U.S. Army Medical Research Institute of Chemical Defense, Aberdeen Proving Ground, Maryland, United States of America; 4 MIT Computer Science and AI Laboratory, Cambridge, Massachusetts, United States of America; 5 School of Systems Biology, George Mason University, Manassas, Virginia, United States of America; Hospital for Sick Children, Canada

## Abstract

Human Serum paraoxonase 1 (HuPON1) is an enzyme that has been shown to hydrolyze a variety of chemicals including the nerve agent VX. While wildtype HuPON1 does not exhibit sufficient activity against VX to be used as an *in vivo* countermeasure, it has been suggested that increasing HuPON1's organophosphorous hydrolase activity by one or two orders of magnitude would make the enzyme suitable for this purpose. The binding interaction between HuPON1 and VX has recently been modeled, but the mechanism for VX hydrolysis is still unknown. In this study, we created a transition state model for VX hydrolysis (VX_ts_) in water using quantum mechanical/molecular mechanical simulations, and docked the transition state model to 22 experimentally characterized HuPON1 variants using AutoDock Vina. The HuPON1-VX_ts_ complexes were grouped by reaction mechanism using a novel clustering procedure. The average Vina interaction energies for different clusters were compared to the experimentally determined activities of HuPON1 variants to determine which computational procedures best predict how well HuPON1 variants will hydrolyze VX. The analysis showed that only conformations which have the attacking hydroxyl group of VX_ts_ coordinated by the sidechain oxygen of D269 have a significant correlation with experimental results. The results from this study can be used for further characterization of how HuPON1 hydrolyzes VX and design of HuPON1 variants with increased activity against VX.

## Introduction

Organophosphorus nerve agents (OPNAs) such as soman, sarin, VR, and VX irreversibly inhibit acetylcholinesterase (AChE). The inhibition of AChE leads to an excess of acetylcholine (ACh) at the neuronal synapse, causing tremors, fasciculations, and eventually death by disruption of cardiac and respiratory function [1]. There are various treatments for OPNA exposure, but these all have significant limitations. Anticholinergics ameliorate the effects of excess ACh [2], but do not remove the nerve agent from the synaptic cleft or restore activity to inhibited AChE. Oximes can be used in conjunction with anticholinergics to reactivate AChE after OPNA exposure, though these compounds are only effective if they are administered prior to dealkylation (“aging”) of the inhibited enzyme. Finally, carbamates can be used to enhance protection against rapidly aging OPNAs, but must be administered prior to exposure [2].

Modified human enzymes designed to rapidly hydrolyze OPNA nerve agents would be ideal treatments for OPNA exposure. These enzymes (“OPases”) could be administered either before or shortly after exposure and would have the benefit of eliminating OPNAs from the bloodstream before they inhibit AChE, rather than simply masking their effects. OPases would also be able to provide a many-to-one effect where a single enzyme molecule could neutralize multiple OPNA molecules.

One enzyme that has been identified as a potential catalytic scavenger of VX is human serum paraoxonase 1 (HuPON1) [3]. HuPON1 is a 355-residue, 43-kDa, calcium-dependent protein that forms a six-fold beta-propeller. HuPON1 contains one “structural” calcium ion which is necessary to maintain the protein's structure and one “catalytic” calcium ion which is necessary for catalytic activity [4]. HuPON1 is synthesized in the liver and is known to bind high-density lipoproteins (HDLs) in the bloodstream [5]. The enzyme is also known to have an inherent level of activity against organophosphates [6], though this is thought to be secondary to its lactonase activity [7], [8]. Native HuPON1 does not have sufficient activity toward OPNAs to be an effective *in vivo* treatment against these compounds. However, it has been speculated that a ten-fold increase in HuPON1's ability to destroy VX could make the enzyme an effective countermeasure against VX [9].

Due to HuPON1's inherent ability to hydrolyze OPNA nerve agents, there has been a great deal of research into understanding HuPON1's OPase mechanism. This work has spanned both experimental [5], [10] and computational [11], [12], [13] methods. Previous research has identified a variety of residues that are believed to be important for substrate interactions (L69, H115, F222, L240, L267, D269, C284, H285, F292, T332, V346, F347) and calcium coordination (E53, D54, N168, N224, D269, N270) [10], [13], [14]. While such “key residues” have been identified, the mechanism by which HuOPN1 hydrolyzes VX remains unknown. Initially, it was hypothesized that H115 and H134 serve as a catalytic dyad where H115 activates a water molecule for attacking VX's phospho-sulfur (P-S) bond [15]. However, it has since been shown that H115 can be mutated to tryptophan without a loss of catalytic activity toward VX [16]. A recent docking and molecular dynamics analysis [11] hypothesized that the active site of HuPON1 has two distinct binding regions – one for lactones and esters, and one for organophosphates, with D269 and/or E53 potentially serving as the catalytic residue for OP hydrolysis. An in-depth docking and molecular dynamics study has also been performed that thoroughly characterized how HuPON1 binds VX [12]. This study demonstrated that VX's lone oxygen atom has a strong preference for binding HuPON1's active site calcium ion, and that VX's hydrophobic tail tends to face outward from HuPON1's active site when VX is bound.

One approach to better understanding the exact mechanism by which HuPON1 hydrolyzes VX is to determine how the enzyme stabilizes the reaction's high energy transition state. Previous studies have demonstrated that the predicted binding energies between transition states and enzymes can be well correlated with experimental data [17], [18]. Additionally, the experimental activities of multiple HuPON1 variants are known. Such information can be utilized to determine which computational binding procedures best predict the experimental activities of HuPON1 variants for VX hydrolysis. This information can then be utilized to both understand the reaction mechanism and increase HuPON1's ability to destroy VX.

In this work, we use transition state docking with a novel clustering approach to determine which transition state binding mechanisms are best correlated to experimental results. Clustering is performed using a novel algorithm that groups bound conformations according to different reaction mechanisms. This grouping procedure eliminates the problem of having multiple predicted binding conformations for a transition state and enables calculation of average binding energies for conformations corresponding to a single reaction mechanism. By comparing the cluster-based average binding energies to experimental enzymatic activities, we are able to determine which computational methods best predict HuPON1 hydrolysis activity toward VX and also gain insight into potential mechanism for this reaction.

## Materials and Methods

### Experimental Characterization of HuPON1 Mutants

Sequences for PON1 mutants were incorporated into expression plasmids by GenScript (Piscataway, NJ, USA). Mutant PON1 protein was expressed via transient infection of 293T cells as described in [16]. Racemic VX was obtained from the U.S. Army Edgewood Chemical Biological Center. Stock solutions were prepared at 0.9 mg mL^−1^ in 5 mM Tris, 10 mM CaCl2 at pH 7.4 and stored at −70°C. A modified Ellman-based colorimetric assay [16] was used to assess hydrolysis activity. Catalytic efficiency (*k_cat_*/K*_M_*) was calculated using the Michaelis-Menten equation with the approximation that the substrate concentration was much less than *K_M_*.

### HuPON1 Mutant Structure Generation

The binding predictions utilized four different structures of the WT HuPON1 enzyme that were generated previously[12]. Briefly, the first of the four structures, the “ITASSER” conformation, is based on a prediction by the I-TASSER server [19], [20] that was subsequently energy minimized in Amber 10 [21]. The second HuPON1 model, the “ITASSER/SMD” conformation, is from a steered molecular dynamics (SMD) simulations that modeled VX binding to the ITASSER structure. The third HuPON1 structure, the “MODELLER” conformation, was generated using the MODELLER software package [22] with independent characterization of HuPON1's disordered loop region. Finally, the “MODELLER/AD4” structure was generated by predicting how VX binds the MODELLER structure using AutoDock4 [23], then simulating the bound complex using molecular dynamics. All four conformations have a backbone structure similar to that observed in the recombinant PON1 structure determined by Harel et al. [14] but have differences in the orientation of HuPON1's active site residues.

Structural models of each HuPON1 variant were generated across all four initial WT HuPON1 models using the SCWRL sidechain replacement program [24]. During sidechain replacement, all residues within HuPON1 were held fixed except for those being mutated. The resulting protein structures were energy minimized using the *sander* program in the Amber10, and the final conformations were saved in PDB format for subsequent docking simulations.

### Transition State Structure Generation

Initial structures of the VX P(+) and VX P(−) stereoisomers were obtained by converting the simplified molecular input line entry specification (SMILES) [25] for VX into three-dimensional coordinates using an online conversion tool made available by the National Cancer Institute (http://cactus.nci.nih.gov/services/translate/). AM1-BCC charges for each VX enantiomer were then calculated using the *antechamber* program from the Amber 10 molecular dynamics suite. Next, transition state models for the P(+) and P(−) VX enantiomers were created through SMD simulations using the *sander* program in Amber10. Initially, each starting conformation of VX was placed in a 10 Å rectangular box of explicit solvent and subjected to 500 steps of quantum mechanical/molecular mechanical (QM/MM) minimization at the PM3-PDDG level. For these simulations, the quantum mechanical potential was applied to the VX atoms, and the standard Amber 10 molecular mechanical potential was applied to all other atoms. The system was then heated from 0.1K to 300K over the course of 50ps followed by 50ps of density equilibration to 1 atmosphere using *sander*. A water molecule located 4–5 Å away from the VX phosphorous atom was then chosen to perform an SN2 attack on the P-S bond. Using steered molecular dynamics, the oxygen atom of this water molecule was moved to a non-reactive distance of 3.6 Å from the VX phosphorus atom. For this simulation, the quantum mechanical potential was applied to both VX and the attacking water molecule, while all other atoms were modeled using the FF99SB force field [26]. Using SMD, the oxygen atom of the selected water molecule was then driven to a distance of 1.6 Å from the phosphorous atom of VX, resulting in the cleavage of VX's P-S bond. The free energy profile was then examined across the reaction coordinate, and the conformation with maximal free energy was extracted as the transition state model for the reaction.

### Molecular Docking

Molecular docking simulations were performed using AutoDock version 4.2 [23]. Structures of the VX P(+) and VX P(−) transition states were prepared using the *prepare_receptor* and *prepare_ligand* scripts from the AutoDockTools package [27] These scripts automatically determined Gasteiger charges [28] for each transition state. AM1-BCC charges for both transition state models were also calculated using the *antechamber* program in Amber10. Docking simulations were then performed across all possible combinations of the HuPON1 structures, VX enantiomers, and charge models. For these simulations, the calcium ions of HuPON1were given a charge of positive two. Additionally, the AutoDock 4, search box size was set to 20 Å with a grid resolution of 0.375 Å. The grid box was centered on the sidechain heavy atoms of residues 222, 224, 240, 285, 292, and 346, which surround the active site of HuPON1. Each docking simulation returned 100 separate binding predictions. Further, each docking simulation was repeated 20 times using different random number generator seeds (resulting in 2000 total predicted structures for each combination being tested). Average binding energies and standard deviations were determined across the 20 independent replicates for each binding simulation.

### Clustering Procedure and Correlation Analysis

Once docking was completed, the docked poses were clustered by their reaction mechanisms. To cluster the bound conformations by reaction mechanism, we developed a novel metric that gauges the alignment of the VX transition state's attacking OH group with key residues in HuPON1's active site. The specific procedure was as follows:

- A vector was first generated that originated at the phosphorus atom of the VX transition state and extended through the attacking oxygen atom. A point (*p_b_*) was then selected along this vector at a fixed distance (d_s_) from the attacking oxygen atom. The distance (d_c_) between *p_b_* and the center of one key atom for one selected residue was determined (*e.g.*, one sidechain oxygen of residue D269). If the measured distance was less than a threshold value (d_t_), then the given bound conformation was indicated as belonging to the reaction mechanism corresponding to the selected residue.
- The process was repeated for additional key atoms and selected residues.

**Figure 1:**
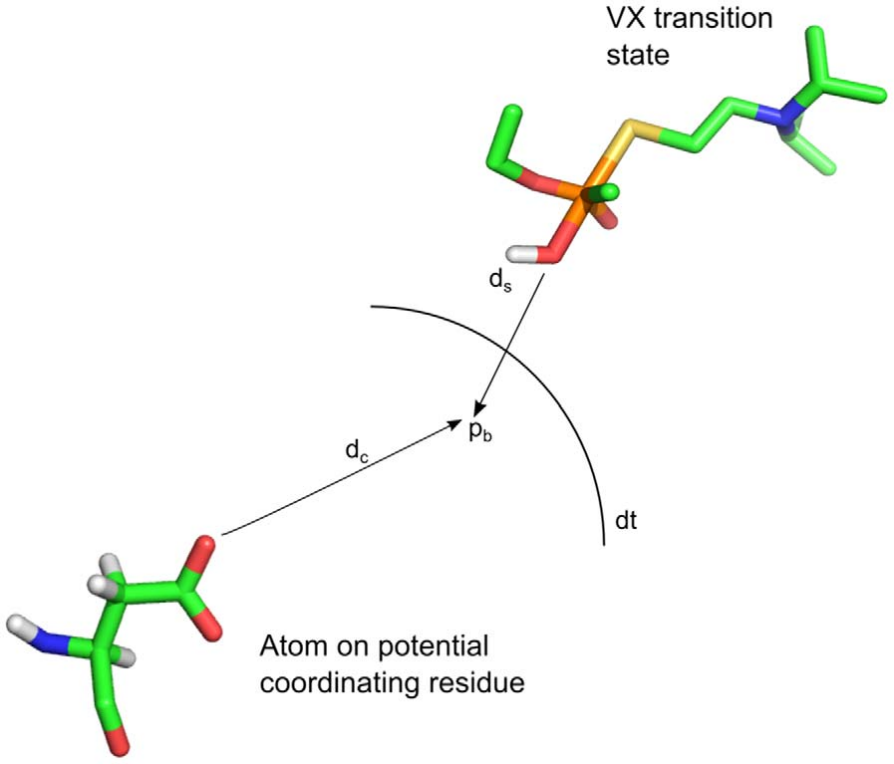
The clustering method used to group conformations by reaction mechanism. The two main parameters, d_s_ and d_t_ are shown.

The above clustering procedure is designed to evaluate if each key atom within HuPON1's active site is in an appropriate position for coordinating the attacking water molecule in VX's transition state. The clustering mechanism is shown graphically in Figure 1.

Using the above algorithm, clusters were determined for side-chain oxygen atoms of E53, D183, D269, and side-chain nitrogen atoms of H285. These atoms were selected because they all can potentially accept a proton from an attacking water molecule. The OH group alignment was also analyzed relative to the active site calcium ion which can potentially stabilize an attacking hydroxide ion.

When performing the cluster analysis, the two main parameters that determine which bound conformations belong to a particular cluster are d_s_ and d_t_. It is unclear *a priori* which values for these parameters will return bound clusters that best match experimental data. Therefore, calculations were performed using multiple combinations of d_s_ and d_t_. The d_s_ values were varied from 1.00 Å to 2.50 Å in 0.25 Å increments and the d_t_ values were varied from 1.25 Å to 3.00 Å in 0.25 Å increments (56 total combinations).

Correlation analyses were performed across all possible combinations of HuPON1 structures, VX enantiomers, transition state charge models, clustering parameters, and reaction mechanisms. For each variant and each binding simulation, the structure that had the lowest binding energy while also matching the current clustering parameters was selected. The best binding energies were then averaged across all 20 replicate binding simulations for each HuPON1 variant. The resulting average values were then utilized to determine Pearson correlation values with experimental enzymatic activities Specifically, correlation values were calculated between the cluster-based average binding energies and -2.3*RTlog(*k_cat_/K_M_*), where *k_cat_/K_M_* represent the experimentally derived enzymatic activities [29]. To avoid spurious results, average binding energies that were based on fewer than 10 data points were discarded. Additionally, correlation values were only considered if they utilized data from at least 20 of the 22 HuPON1 variants.

## Results and Discussion

### Experimental characterization of HuPON1 mutants

Table 1 shows the experimental VX hydrolysis activities for the HuPON1 mutants examined in this study. Wildtype HuPON1 (WT) has an average k_cat_/k_M_ of 59.8, calculated from two separate experiments. Among the variants tested, only H115W has higher activity than WT with an average *k_cat_/K_M_* of 130.0[16]. The remaining variants all have lower activities than WT with average *k_cat_/K_M_* values ranging from 2 to 57.

**Table 1:** HuPON1 variants analyzed in this study.

Mutant	*k_cat_/K_M_* (min^−1^ mM^−1^)	Mutant	*k_cat_/K_M_* (min^−1^ mM^−1^)
WT	59.8±11.1	F292W	19.4±0.4
L69V	2.5±0.4	V346A	5.9±0.5
Y71A	5.0±2.1	V346W	18.3±0.1
Y71D	3.0±0.9	M1 (H115W,V346I,F347L)	14.6±3.6
Y71E	1.5±0.3	M2 (H115Y,F222W,F292W)	3.7±0.6
Y71K	0.2±0.03	M3 (F222W, L240F, F292L)	22.1±0.2
Y71N	0.5±0.6	M4 (F222W, L240I, F292W)	0.9±0.1
Y71R	0.3±0.02	M5 (F222Y, F292A)	0.3±0.04
H115W	130.0±15.3	M6 (L240A,F292L)	4.5±0.2
H115Y	15.0±5.2	M7 (F292A, V346W)	5.3±0.1
F292A	1.1±0.42	M8 (F292W, V346W)	34.9±5.6

± indicates standard deviation over three experiments. Please note that deviations do not include errors in protein measurement.

### Transition state model for VX hydrolysis in water

**Figure 2:**
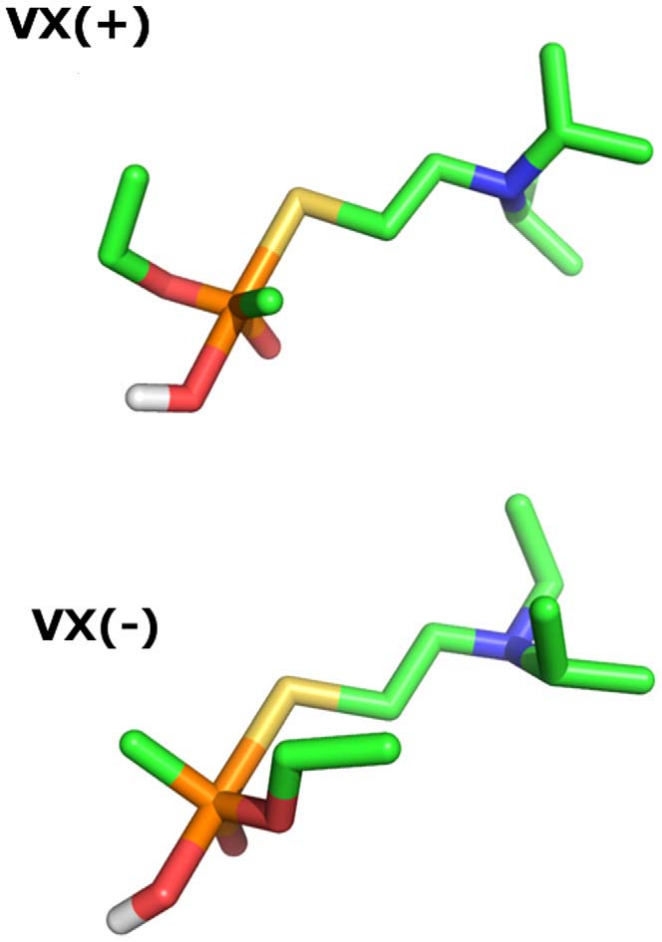
The transition state conformations for VX P(+) and VX P(−). The structures were generated with the QM/MM capabilities of the AMBER molecular dynamics suite [21].

Figure 2 shows the structures of the transition state models of VX(+) and VX(−) [abbreviated VX_ts_ P(+) and VX_ts_ P(−), respectively] used in docking simulations. The two stereoisomers of the TS share many similarities. Both have the attacking water's oxygen atom and VX's sulfur atom in-line with the phosphorous atom of VX while the remaining constituents on the phosphorus atom exhibit a trigonal planar geometry. The interatomic distances between the breaking P-S bond are 2.5 Å in VX_ts_ P(−) and 2.4 Å in VX_ts_ P(+), while the nascent O-P bond length is 2.0 Å for VX_ts_ P(−) and 1.9 Å for VX_ts_ P(+). It should be noted that, due to limitations in the AutoDock software, one of the hydrogen atoms had to be removed from the attacking water in order for the molecule to be prepared for docking.

### Computational Binding Predictions

Table 2 lists the binding procedures and clustering parameters whose predicted average binding energies had the highest correlation with experimental *k_cat_/K_M_* values. As the table shows, the top methods all utilize the ITASSER/SMD structure of HuPON1, the VX_ts_ P(−) transition state model, and the D269-based hydrolysis mechanism (*i.e.*, with the attacking water aligned toward the side-chain of D269). This trend holds across the first 40 methods with the highest correlation values (data not shown). The only difference between these 40 methods is that they have slightly different values of d_s_ and d_t_. The fact that these prediction methods all have similar correlation scores indicates that the clustering results are robust across a range values for d_s_ and d_t_. The 41^st^ best correlated method also has a very similar setup to the 40 top correlated methods and uses the ITASSER/SMD structure with the D269 mechanism. The only difference is that the 41^st^ best correlated method uses the VX_ts_ P(+) transition state model [rather than VX_ts_ P(−)]with d_s_ and d_t_ values of 1.25 Å and 2.00 Å respectively. This approach returns a correlation of 0.657 across the 22 experimental samples with a corresponding p-value of 6.51×10^−4^.

**Table 2:** Top scoring parameters by correlation analysis.

Coordinating Group	d_s_	d_t_	HuPON1 Structure	TS Model	Charge Model	Corr	p-value	Data Points
D269	2.00	2.25	ITASSER/SMD	VX_ts_ P(−)	Gast.	0.767	3.17E-05	22
D269	1.75	2.25	ITASSER/SMD	VX_ts_ P(−)	Gast.	0.765	3.35E-05	22
D269	2.25	2.25	ITASSER/SMD	VX_ts_ P(−)	Gast.	0.762	3.71E-05	22
D269	2.50	2.25	ITASSER/SMD	VX_ts_ P(−)	Gast.	0.761	3.88E-05	22
D269	2.00	2.00	ITASSER/SMD	VX_ts_ P(−)	Gast.	0.760	4.04E-05	22
D269	2.25	2.00	ITASSER/SMD	VX_ts_ P(−)	Gast.	0.759	4.29E-05	22
D269	1.25	2.25	ITASSER/SMD	VX_ts_ P(−)	Gast.	0.754	5.01E-05	22
D269	1.00	2.25	ITASSER/SMD	VX_ts_ P(−)	Gast.	0.754	5.11E-05	22
D269	1.50	2.25	ITASSER/SMD	VX_ts_ P(−)	Gast.	0.750	5.82E-05	22
D269	2.50	2.50	ITASSER/SMD	VX_ts_ P(−)	Gast.	0.750	5.82E-05	22

The p-values for the top performing clusters indicate that the best correlations are not due to random chance. Let *C_x_* represent a single correlation score with an associated p-value *p_x_*. Further, let ***A*** represent a set of *N* correlation values obtained by random chance. Since the p-value represents the probability that a random correlation value will be more extreme than *C_x_*, the probability that all N samples in ***A*** will be less than or equal to *C_x_* is



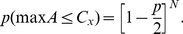
(1)


The binding energy predictions were calculated across four HuPON1 structures, two transition state models, two charge models, and five hydrolysis mechanisms, giving a total of 80 possibilities (*N* = 80). The seven possible values for d_s_ and eight possible values for d_t_ are not included in the total combinations since the correlation results indicated the cluster-based binding energies are not independent across these two parameters. Plugging the values for the top correlated method in Table 2 (correlation  = 0.767, *p* = 3.17×10^−5^) into Equation 1 gives a 99.87% probability that a higher correlation score would not be obtained by chance across 80 random correlation values. This indicates a high level of certainty that the best correlation value is not due to random chance. Even the 41^st^ best method which uses VX_ts_ P(+) has a 97.0% chance of being better than 80 random correlation values. Across all possible combinations of binding energy calculations, only those methods that utilize the ITASSER/SMD structure of HuPON1 in combination with the D269 mechanism and either VX_ts_ P(−) or VX_ts_ P(+) have greater than 95% probability that their correlation values will be higher than 80 random correlation values. Thus, across the methods tested, only those that are based on the D269 hydrolysis mechanism have significant correlation with the experimental data. Higher correlation values are observed with VX_ts_ P(−) than VX_ts_ P(+). Recent experimental data indicates that WT HuPON1 is only capable of hydrolyzing the P(+) enantiomer of VX [30]. The fact that VX_ts_ P(−) returns higher correlation values could be due to inaccuracies that are inherent in static binding procedures. However, the p-values still show that that the correlation values are significant for both VX_ts_ P(+) and VX_ts_ P(−).

An interesting point about the correlation results is that only the ITASSER/SMD structure of HuPON1 returns statistically significant correlation values. This is likely due to the different ways in which the HuPON1 structures were constructed. The ITASSER/SMD conformation of HuPON1 was generated by running steered molecular dynamics simulations that drove VX into HuPON1's active site. This process can simulate how an enzyme's structure is modified to accommodate a substrate during initial binding [12] and likely provides a better structural model for static binding studies with the transition state structure (since the active site structure has already been modified to accommodate the substrate). In contrast to this, both the ITASSER and MODELLER structures were generated through homology modeling without accounting for the ligand being present in HuPON1's active site. Thus, these two HuPON1 structures are likely in a non-ideal conformation for interacting with the substrate. For the MODELLER/AD4 structure, HuPON1's conformation was determined by running molecular dynamics (MD) with VX bound in the active site. Given that the MD simulations enable repositioning of active site residues to better interact with the ligand, it seems reasonable that this structure could better accommodate the TS models and provide higher correlation scores. Indeed, when looking at correlation score, binding predictions that utilized the MODELLER/AD4 structure do outperform methods using the ITASSER and MODELLER structures. However, none of the correlations for the MODELLER/AD4, ITASSER, or MODELLER structures have high enough p-values to be considered statistically significant at the 95% level (using Equation 1 with *N* = 80).

**Figure 3:**
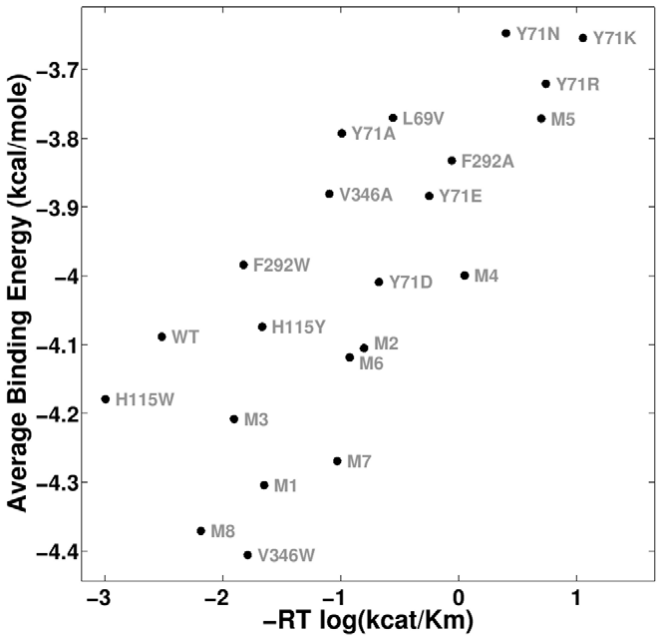
Scatter plot of experimental vs. predicted activity for the top-scoring set of variables (D269 mechanism, d_s_ = 2.00 Å, d_t_ = 2.25 Å, ITASSER/SMD structure, VX_ts_(−) enantiomer, and Gasteiger charges). This dataset has a Pearson correlation of 0.767 (p<10^−4^)

Figure 3 shows that an excellent match is obtained between the observed catalytic efficiency (*k_cat_/K_M_*) of variants in Table 1 and the predicted binding energies for these same variants. The relatively high correlation value for these data points, coupled with the corresponding p-value, indicate that the computational method can predict which HuPON1 variants are more likely to have better VX hydrolysis activity. The deviations from a perfect linear fit in Figure 3 could stem from various factors. These include errors in the binding predictions (as discussed above) and errors in the experimental data points.

**Figure 4:**
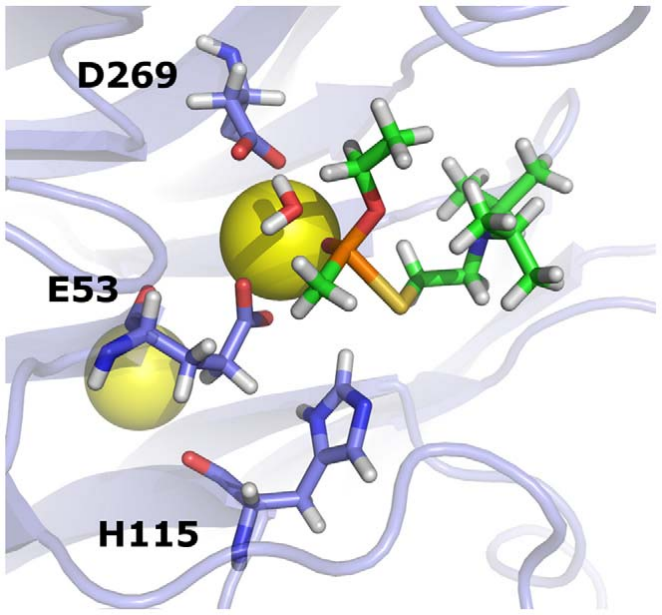
Predicted binding conformation of WT HuPON1 and VX_ts_(+) according to the D269-based hydrolysis mechanism. The specific binding method utilized the ITASSER/SMD structure of HuPON1 with d_s_ = 1.25 Å and d_t_ = 2.00 Å. The structure has been energy minimized in Jaguar [31] using the OPLS energy function [32] with distance constraints on the breaking P-S bond and the nascent O-P bond. A full water molecule was utilized as the nucleophilic group for proper charge modeling.

Figure 4 illustrates some key features of the binding methods with significant correlations. Here, the VX_ts_ P(+) model is bound to the ITASSER/SMD structure of HuPON1 according to the D269-based hydrolysis mechanism. The attacking hydroxide ion has been converted to a full water for proper charge modeling, and the entire structure has been minimized using the OPLS molecular mechanical energy function with distance constraints on the breaking P-S bond and nascent O-P bond (to preserve the transition state geometry). The resulting structure illustrates many features that were predicted in previous binding studies with HuPON1 and VX. Specifically, the lone oxygen from VX is found to form a direct interaction with the HuPON1 active site calcium while the hydrophobic tail of VX points outward from the enzyme active site. Figure 4 also illustrates that the attacking water is oriented so its oxygen atom is near the phosphorus atom of VX and one of its hydrogen atoms is near a side-chain oxygen of D269. Interestingly, the water molecule's other hydrogen atom is found adjacent to a sidechain oxygen of residue E53. This raises the possibility that D269 and E53 may both help stabilize a water molecule while it attacks VX's phospho-sulfur bond, with each residue using one of its sidechain oxygen's to help stabilize a hydrogen on the attacking water molecule.

### Conclusion

We have utilized existing experimental data, along with quantum mechanical/molecular mechanical simulations and transition state docking to further understand the mechanism of VX hydrolysis by HuPON1. Through a novel clustering procedure, we examined which orientations of VX's transition state within HuPON1's active site returned predicted binding energies that are best correlated to known experimental activities. In doing this, we identified computational binding procedures that are well correlated with experimental data for 22 HuPON1 mutants. Across these various binding procedures examined, statistically significant correlations were only obtained when the attacking oxygen atom was aligned with the sidechain of D269. Further computational studies for this predicted binding conformation revealed that residues E53 and D269 may both help stabilize protons of a water molecule whose oxygen atom is attacking the phospho-sulfur bond of VX. These findings may be used to guide further computational analysis of the hydrolysis of VX by HuPON1, as well the design of a HuPON1 mutant with increased activity against VX.
